# Multiscale and Hierarchical Feature-Aggregation Network for Segmenting Medical Images

**DOI:** 10.3390/s22093440

**Published:** 2022-04-30

**Authors:** Nagaraj Yamanakkanavar, Jae Young Choi, Bumshik Lee

**Affiliations:** 1 Department of Information and Communications Engineering, Chosun University, Gwangju 61452, Korea; nagarajpy@chosun.ac.kr; 2 Division of Computer & Electronic Systems Engineering, Hankuk University of Foreign Studies, Yongin 17035, Korea; jychoi@hufs.ac.kr

**Keywords:** convolutional neural network, medical-image segmentation, feature fusion

## Abstract

We propose an encoder–decoder architecture using wide and deep convolutional layers combined with different aggregation modules for the segmentation of medical images. Initially, we obtain a rich representation of features that span from low to high levels and from small to large scales by stacking multiple *k* × *k* kernels, where each *k* × *k* kernel operation is split into *k* × 1 and 1 × *k* convolutions. In addition, we introduce two feature-aggregation modules—multiscale feature aggregation (*MFA*) and hierarchical feature aggregation (*HFA*)—to better fuse information across end-to-end network layers. The *MFA* module progressively aggregates features and enriches feature representation, whereas the *HFA* module merges the features iteratively and hierarchically to learn richer combinations of the feature hierarchy. Furthermore, because residual connections are advantageous for assembling very deep networks, we employ an *MFA*-based long residual connections to avoid vanishing gradients along the aggregation paths. In addition, a guided block with multilevel convolution provides effective attention to the features that were copied from the encoder to the decoder to recover spatial information. Thus, the proposed method using feature-aggregation modules combined with a guided skip connection improves the segmentation accuracy, achieving a high similarity index for ground-truth segmentation maps. Experimental results indicate that the proposed model achieves a superior segmentation performance to that obtained by conventional methods for skin-lesion segmentation, with an average accuracy score of 0.97 on the ISIC-2018, PH2, and UFBA-UESC datasets.

## 1. Introduction

Medical-image segmentation, such as tooth segmentation [1] and skin-lesion segmentation [2], is a common step in the analysis of medical images. The main objective of medical-image analysis is to serve as an efficient diagnosis and treatment tool to radiologists and physicians [3]. Medical imaging, e.g., X-rays, computed tomography, and magnetic resonance imaging (MRI), can nondestructively provide anatomical and functional information about diseases and abnormalities inside the body. However, there are several challenges associated with the automatic segmentation of medical images. Medical images can be acquired using various protocols, and they often have a low contrast and inhomogeneous appearance, making them difficult to segment [4]. Additionally, some structures are characterized by large variations in scale and shape, such as skin lesions in dermoscopic images, which makes it difficult to construct prior models of their shapes in advance [5]. Challenges associated with panoramic X-ray images include variations in teeth among patients and spaces between missing teeth [6]. To achieve a better segmentation performance, automated segmentation techniques should consider the scale and position of the target.

In recent years, various models have been proposed for segmenting medical images using deep convolutional neural networks (CNNs) [7]. CNNs have better representation abilities than traditional approaches, allowing them to automatically learn the most useful features from large datasets compared to traditional machine-learning approaches. At present, most CNNs suffer from the following issues. First, the convolutional-layer design uses shared weights at different spatial positions, resulting in a lack of spatial awareness. This degrades the performance for targets with variable shapes and positions—particularly small targets. Second, CNNs generally utilize numerous redundant feature channels. Third, CNNs do not always recognize the most appropriate scale for identifying the segmentation features of an image [8,9]. Finally, most existing CNNs do not have a straightforward way to explain their decisions and are used in a black-box manner because they are nested nonlinear structures, which makes their application in clinical decision-making difficult.

Hence, the goal of the study in this paper is to design a CNN architecture for the task of medical image segmentation that uses novel feature aggregation modules (which solve the first problem) and guided skip-connections (which solve the second problem). The proposed architecture can also provide accurate border localization and delineation through the proposed guided block. Furthermore, the proposed method using residual-based feature aggregation modules shows an improved performance, with a significant reduction in the number of trainable parameters (mitigating the third problem). To address the aforementioned problems, Ronneberger et al. [10] introduced the U-Net architecture for the segmentation of neural structures in electron microscopic recordings and cell identification in microscopic images. Adiga et al. [11] developed an M-Net architecture for fingerprint image inpainting and denoising by adding the left-leg path to the encoder side and the right-leg path to the decoder side. However, the M-Net architecture can lose local information when complete images are provided as inputs. Zhou et al. [12] proposed a U-Net++ architecture based on encoders and decoders for medical-image segmentation. Each block in the network is connected through a series of densely nested skip paths. Gu et al. [13] developed a context encoder network (CE-Net) for medical-image segmentation that utilizes a pre-trained ResNet block in the feature encoder. However, CE-Net has the drawback that the use of dense connections in the network leads to an increase in the number of parameters. In [14], the M-SegNet architecture was used for the segmentation of brain tissues in MRI. The global attention technique is used in the decoder stage to capture rich contextual information by combining local features with their global dependencies. Jin et al. [15] proposed a residual attention-aware UNet (RA-UNet) architecture for liver and tumor segmentation using a two-dimensional (2D) network followed by a three-dimensional (3D) network. Initially, the input image is fed into a 2D boundary extraction network to segment only the liver region. The 2D outputs are stacked to form the input for the 3D network, which produces tumor-segmentation maps. The overall RA-UNet involves training the 2D network, followed by training the 3D network. This multiple-model training method created a computationally expensive network. Mohammed et al. [16] introduced a contextual multiscale multilevel network (CMM-Net) for medical-image segmentation. In CMM-Net, a pyramid pooling module (PPM) is combined with dilated convolution to extract multiscale information for biomedical-image segmentation. The PPM is used to generate global multilevel information at every encoder layer, whereas dilated convolutions learn various spatial scales of the target with minimal resolution loss. Fabian et al. [17] introduced the nnU-Net architecture for biomedical-image segmentation. In nnU-Net, the first 3D U-Net is trained on downsampled 3D images, and the segmentation result is then upsampled and passed to a second 3D U-Net trained on patches at the full resolution. The nnU-Net can handle large disparities in dataset properties and diversity in target structures. However, nnU-Net was designed with a focus on the training process rather than algorithm improvements, leading to suboptimal segmentation performance.

To overcome these limitations, we developed a novel multiscale and hierarchical feature-aggregation network for the segmentation of medical images. Our objective was to improve the fusion of semantic and spatial information for the segmentation of medical images by aggregating layers. We introduce two aggregation structures—multiscale feature aggregation (*MFA*) and hierarchical feature aggregation (*HFA*)—to aggregate deep layers. The *MFA* module gradually accumulates and enriches the representations of features, and the *HFA* structure repeatedly and hierarchically merges the features to learn richer feature combinations. In addition, guided skip connections facilitate the transfer of features between the encoder and the decoder, ensuring the recovery of the spatial information lost during downsampling. The proposed approach significantly outperformed recently developed medical-image segmentation methods in multiple evaluation metrics. The main contributions of this study are summarized as follows:

▪ The proposed model was designed with a multiscale and hierarchical feature-aggregation network to better fuse feature information for the segmentation of medical images.
▪ Guided skip connections from the encoder block to the decoder block are used to improve the segmentation accuracy and the convergence of deep neural networks.
▪ The proposed approach has a good generalization ability according to the results of comparisons with state-of-the-art methods for different challenging tasks involving skin-lesion and tooth segmentation.

The remainder of this paper is organized as follows. Section 2 discusses related works. Section 3 describes the proposed method in detail and explains its architecture. The experimental setup, comparative studies, and a comprehensive analysis are presented in Section 4. Finally, Section 5 concludes the paper.

## 2. Related Work

### 2.1. Multiscale Networks

In recent studies, the performance of neural networks for multiple semantic segmentation tasks was improved via multiscale feature fusion. Several methodologies have been proposed to encode contextual information at different scales [18]. As an alternative to the aforementioned encoder–decoder structure, image pyramid structures [19,20] are frequently used to obtain different scales of objects in the network. Further, the dilated or atrous spatial pyramid pooling (ASPP) convolutions employed in a parallel or cascade design help to expand the network receptive field without demanding additional network parameters [21]. ASPP modifies the atrous convolution simultaneously in relation to spatial pyramid pooling to obtain features on arbitrary scales [22]. In addition, deformable convolution enhances atrous convolution by increasing the number of sampling locations [23]. Furthermore, maintaining a high-resolution image representation is essential for segmentation networks to capture spatial information and generate accurate segmentation maps. The purpose of multiscale fusion is to exchange low- and high-resolution features during the segmentation process, instead of recovering these representations from low-level representations [24]. Wang et al. [25] showed that such a feature transfer strategy can increase the feature resolution, thus increasing the spatial accuracy of segmentation maps.

### 2.2. Skin-Lesion Segmentation

Researchers have introduced several deep-learning frameworks for the detection and segmentation of skin lesions. In [26], multistage fully convolutional networks were proposed for skin-lesion segmentation. In multistage processing, localized coarse appearances are initially learned, and specific boundary characteristics are learned in the later stages. Yuan et al. [27] used a leveraging 19-layer deep CNN method for skin-lesion segmentation. Furthermore, the authors compared the results obtained using different input sizes, optimization methods, augmentation strategies, and loss functions [12,16,17]. In [28], the fully convolutional method was introduced for the multiclass segmentation of dermoscopic images. This was the first study in which a multiclass segmentation method was used to separate melanocytic nevus, melanoma, and seborrheic keratosis from a single skin lesion. Vesal et al. [29] introduced a two-stage CNN-based approach for the detection and segmentation of skin lesions. The first stage uses a faster region-based CNN for multitask learning, which improves the identification accuracy for image lesions, whereas the second employs a region proposal network, which generates multiple bounding boxes for each image. Mohammed et al. [30] used a convolutional network for skin-lesion segmentation that learned the full-resolution features of each pixel from dermoscopic images.

### 2.3. Tooth Segmentation

Recently, numerous studies have been performed on tooth segmentation, as the diagnosis in dentistry is based on the analysis of teeth and surrounding tissues. In traditional image-segmentation approaches [31,32], dental information is extracted from a single type of radiograph. However, these methods depend on well-designed manual features and do not provide sufficient generalization. In recent years, deep-learning techniques have increased the accuracy of dental segmentation under various conditions, e.g., cavities, bone loss, and hidden dental structures [33]. Generally, deep-learning mechanisms for image segmentation are based on end-to-end learning; both training and inference are performed via dense feedforward computing, as well as backpropagation, to learn the entire image at once. Jader et al. [34] were the first to employ panoramic radiography to segment and detect teeth without numbering. In [35], deep learning was first used for the detection and counting of teeth in panoramic X-ray images.

In deep learning, most CNN networks can extract the features of convolution kernels of different sizes. During the encoding process, the CNN networks increase the depths of the feature maps while reducing the spatial dimension, whereas, during the decoding process, these low-dimension feature maps are up-sampled to recover the output segmentation maps. A CNN uses larger kernels when the pixels are homogeneous and global, whereas a smaller kernel is preferred when the pixels are fine and detailed. An individual *k* × *k* kernel is not sufficient for extracting all the necessary information from the CNN networks. With an increase in the kernel size, the number of learnable parameters increases, increasing the risk of overfitting [36]. Increasing the scale of deep neural networks is the most effective technique to improve performance. This entails an increase in the number of network layers and units at each level, as well as increasing the depth and width of the architecture [36]. In [10,11,12,13,14,15,16,17], medical-image segmentation using conventional methods has limitations. For example, the U-Net [10] model reduces the feature resolution for abstract feature representations through convolution striding and pooling operations. M-Net [11] achieves a tradeoff between speed and the maintenance of high-resolution feature maps. Conventional methods [13,14,15,16,17] have more learnable parameters, which increases the computational complexity. Moreover, these conventional methods use a series of convolutional filters of 3 × 3 and 5 × 5 kernels, which are used for to train a large amount of labeled data, with an increase in memory requirements for deeper networks. To overcome these limitations, we developed a hierarchical and multiscale feature-aggregation network for the segmentation of medical images. During the *MFA* process, features are accumulated, enriching the feature representation. In the *HFA* process, the features are merged repeatedly and hierarchically to generate richer feature combinations. Furthermore, guided skip connections transmit information from the encoder to the decoder, thereby restoring the spatial information that is lost during downsampling.

## 3. Proposed Methodology

As discussed in Section 1, despite their promising results, conventional methods for segmenting medical images suffer from significant drawbacks, including the loss of local information and their high computational complexity. To solve these problems, we proposed a multiscale *HFA* method for medical-image segmentation. The following subsections present (i) an overview of the proposed method and (ii) the proposed feature-fusion architecture.

### 3.1. Overview of Proposed Method

**Figure 1:**
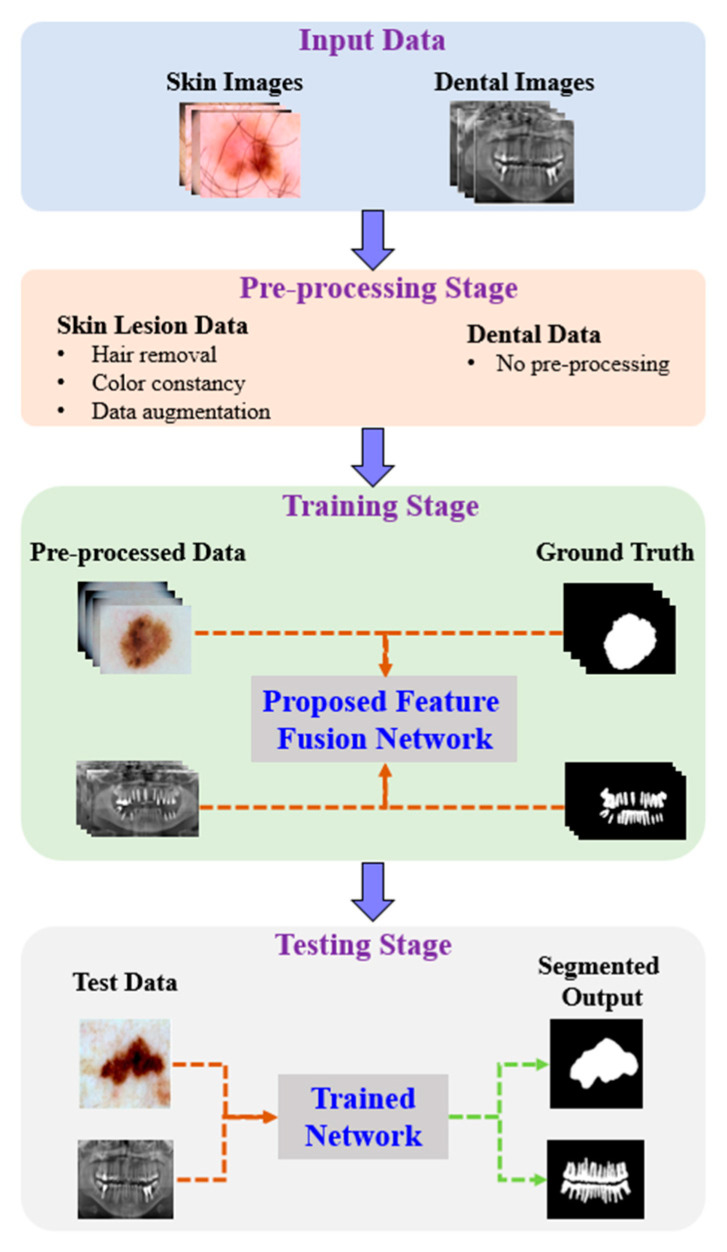
Pipeline of the proposed method.

**Figure 2:**
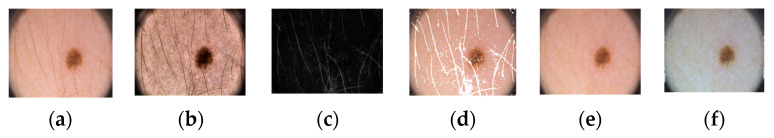
Preprocessing steps: (**a**) original image, (**b**) contrast-enhanced image, (**c**) hair mask, (**d**) hairless image with removed pixel information, (**e**) hairless image, and (**f**) gray-color image.

Figure 1 shows the proposed architecture. As shown, the proposed method comprises four phases: input data, preprocessing, training, and testing. In the input phase, the ISIC-2018 [37] and PH2 [38] datasets are used for skin-lesion segmentation of dermoscopic images, and the UFBA-UESC [39] dataset was used for tooth segmentation of X-ray images. Furthermore, preprocessing was performed on the skin lesion to improve the quality of the input images. Before training the model, the skin-lesion datasets were preprocessed to improve the quality of images using morphology-based inpainting and gray-color constancy algorithms [40,41]. Figure 2 shows the preprocessing stages of dermoscopic images for skin hair removal and conversion to gray-color images. First, contrast-limited adaptive histogram equalization (CLAHE) was employed to improve the visibility of the dermoscopic images. As shown in Figure 2c, the curvilinear mask was extracted using the curvilinear object detector, which employs soft color top-hat transforms [42]. The difference between Figure 2b,c produced a hairless image with pixel information removed, as shown in Figure 2d. The inpainting method utilized morphological operations to rewrite information from the removed pixels, resulting in a hairless image, as shown in Figure 2e. After hair removal, the color of the dermoscopic images was normalized using the gray shade algorithm [41] before training and testing to improve the segmentation results. Furthermore, the datasets were augmented to increase the amount of training data. Images were center-cropped, rotated randomly by 90°, subjected to grid distortion with a limit of 0.3, and flipped horizontally and vertically for data augmentation.

### 3.2. Proposed Feature-Fusion Architecture

**Figure 3:**
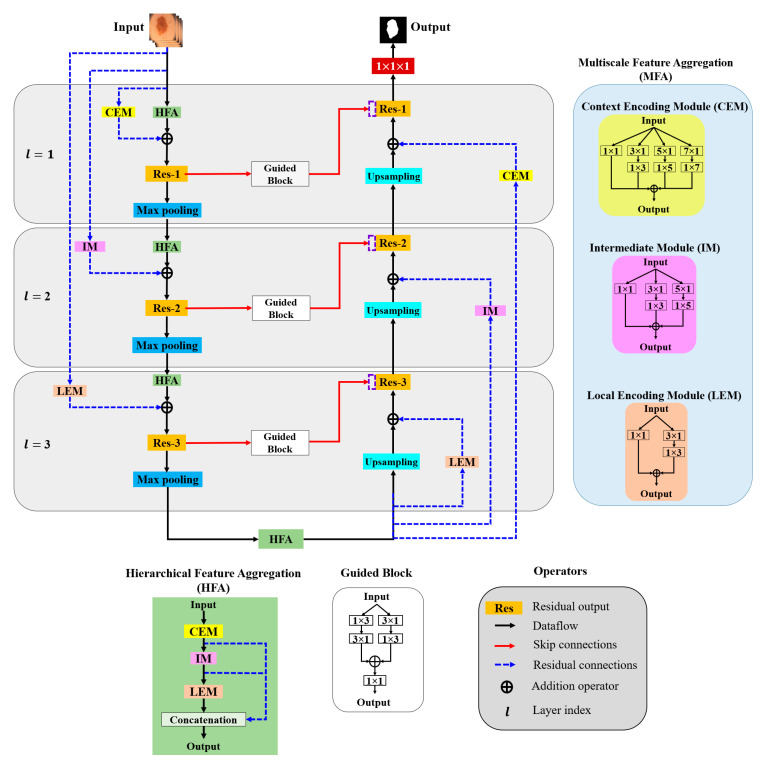
Overall architecture of the proposed model.

In this paper, aggregation is defined as the combination of different convolution kernels throughout the network layers, and an architecture for the effective aggregation of feature information across different scales is proposed. Figure 3 presents a schematic of the proposed method. As shown, *MFA* and *HFA* structures are used for effective feature extraction via an end-to-end network layer. 

The *MFA* structure focuses on fusing spatial convolution kernels with different scales, and the *HFA* merges features from all the *MFA* blocks. The *MFA* structure follows the basic stacking of multiscale spatial kernels (context encoding module (*CEM*), intermediate module (*IM*), and local encoding module (*LEM*)) with the respective network layers. The *HFA* employs an identity connection that crosses and merges MFA blocks to aggregate different levels of feature representation. Furthermore, *MFA*-based long residual connections are used to stabilize the gradient updates along the aggregation paths. In addition, the guided block with multilevel convolution provides effective attention to the features passed from the encoder to the decoder to recover spatial information. The proposed architecture consists of three components: (a) *MFA*, (b) *HFA*, and (c) encoder and decoder blocks. A detailed description of each component is presented in the following subsection.

#### 3.2.1. Multiscale Feature Aggregation (*MFA*)

The *MFA* module consists of the stacking of multiple *k* × *k* convolution kernels, and each *k* × *k* kernel operation is split into a *k* × 1 kernel, followed by a 1 × *k* kernel. The *MFA* is divided into three modules according to the type of feature extraction: the *CEM*, *IM*, and *LEM*.

- Context Encoding Module (*CEM*)

The *CEM* is designed to capture local and contextual information from the input data using both smaller (3 × 3) and larger kernels (5 × 5 and 7 × 7). In each of these *k* × *k* kernels, the operation is decomposed into two smaller spatial kernels: a *k* × 1 kernel followed by a 1 × *k* kernel. This method of splitting kernels produces a two-layer convolution structure that increases the depth of the feature space. Furthermore, the *CEM* consists of a stack of multiple kernels (k = 1, 3, 5, 7), which increases the width of the network layer. The outputs of all these kernels are combined to form a *CEM* output. This causes the *CEM* to increase the width and depth of the network layer and learn global abstract structures. Considering $x^{\left[l\right]}$ as the input sample with network layer index l, the convolutional output *CEM* ($CE_{o}$) is defined as follows:

(1) $$CE_{o}=\sum_{k=1,3,5,7}\left\{\left(\left(x^{\left[l\right]}*w_{k\times 1}^{\left[l\right]}\right)*w_{1\times k}^{\;\left[l\right]}+b^{\left[l\right]}\right)\right\},$$

where $w_{k\times 1}^{\left[l\right]}$ is the weight associated with kernel size *k*, $b^{\left[l\right]}$ is the bias term, and ∗ represents the convolution operation with rectified linear unit (ReLU) activation.

- Intermediate Module (*IM*)

In the *IM*, the network layer is kept narrow and deep to extract local and complex features. Furthermore, the IM contains only 3 × 3 and 5 × 5 convolution kernels; that is, the 3 × 3 kernel operation is divided into 3 × 1 and 1 × 3 kernels and the 5 × 5 kernel operation is divided into 5 × 1 and 1 × 5 kernels, and the outputs are added elementwise to form the *IM* output *(*$IM_{o}$), which is expressed as follows:

(2) $$IM_{o}=\underset{k=1,3,5}{\sum }\left\{\left(\left(x^{\left[l\right]}*w_{k\times 1}^{\left[l\right]}\right)*w_{1\times k}^{\;\left[l\right]}+b^{\left[l\right]}\right)\right\}.$$

- Local Encoding Module (*LEM*)

The *LEM* includes only a 3 × 3 kernel operation that is divided into 3 × 1 and 1 × 3 kernels to extract local and fine detailed features. The *LEM* output ($LE_{o}$) is computed as follows with *k* = 1 and 3:

(3) $$LE_{o}=\underset{k=1,3}{\sum }\left\{\left(\left(x^{\left[l\right]}*w_{k\times 1}^{\left[l\right]}\right)*w_{1\times k}^{\;\left[l\right]}+b^{\left[l\right]}\right)\right\}$$

#### 3.2.2. Hierarchical Feature Aggregation (*HFA*)

The *HFA* block combines all the *MFA* modules to preserve and combine the feature channels. To combine multiple *MFA* blocks, short identity residual connections are used, because they are advantageous for the assembly of very deep networks. As shown in Figure 3, the deep branching structure of the *HFA* employing multiple *MFA* blocks connected through short residual paths diminishes gradient vanishing problems along the aggregation paths and is defined as follows:

(4) $$HFA=concatenate\;\left[CE_{o},\;IM_{o},LE_{o}\right].$$

#### 3.2.3. Encoder and Decoder Blocks

The general structures of the *MFA* and *HFA* are employed in the encoder and decoder paths. The encoder is a sequence of convolution kernels and downsampling operators used to extract high-order features. In contrast, in the decoder, these features are decoded by upsampling operators to generate segmentation masks. We propose an *HFA* as the backbone of the encoder path, providing multiscale discriminative feature extraction. Furthermore, the encoder and decoder paths are coupled with different residual connections corresponding to different network layers. As this residual connection traverses from the input layer to the deeper network layers, we denote them as “long residual connections” (blue dotted line in Figure 3). As initial network layers pose generic context details, the deeper stages are semantic and local. Thus, to obtain layer-specific feature information, we present the *CEM*-based residual connections at the initial network layers (l = 1). Furthermore, the second network layer (l = 2) is designed with an IM-based residual connection. Similarly, the third network layer is integrated with an *LEM*-based residual connection. The *CEM*-, *IM*-, and *LEM*-based residuals are employed with stride convolutions to maintain the dimensions that are proportional to the encoder and decoder paths. All the feature-aggregation blocks in the initial network layer (l = 1) have an input filter size of 32, a filter size of 64 in the second network layer (l = 2), and have a filter size of 128 they in the third network layer (l = 3), respectively. In summary, each encoder layer consists of a sequence of an *HFA* block coupled with the respective *MFA*-based long residual connection, followed by 2 × 2 max pooling. Similarly, 2 × 2 upsampling is integrated with the *MFA*-based long residual connection at the decoder path. The *MFA*-based long residual connection from shallower to deeper layers progressively aggregates multiscale feature information and enhances feature representation. The process of aggregation begins at the shallowest, largest scale and then progressively merges into deeper, smaller scales. In this manner, deep features are improved as they are distributed through different stages of aggregation. In addition, the guided block with multilevel convolution (3 × 1 followed by 1 × 3 and 1 × 3 followed by 3 × 1) provides effective attention for the features copied from the encoder to the decoder, forming guided skip connections. These guided skip connections use both higher- and lower-resolution feature information while performing upsampling operations and help learn and refine misaligned boundaries simultaneously. In the final step, the decoder layer output is passed to the 1 × 1 convolutional layer with sigmoid activation functions. These layers function as classifiers, independently determining the probability of each pixel belonging to there background or foreground.

## 4. Experimental Results

Extensive comparative experiments were conducted to evaluate the performance of the proposed method and compare it with state-of-the-art methods. In addition, an ablation study was performed to highlight the importance of each architectural module in the proposed method. 

### 4.1. Datasets

#### 4.1.1. Skin-Lesion Dataset

We used the ISIC-2018 [37] and PH2 [38] datasets for our experiments. In the experiments, the augmented ISIC-2018 and PH2 datasets were composed of 15,564 and 1200 images, respectively, with their associated ground-truth binary masks. The ISIC-2018 dataset included 15,564 images, which were partitioned into three categories: training (9340 images), validation (3112 images), and testing (3112 images). Of the 1200 images in the PH2 dataset, 960 were used for model training, 120 were used for validation and the remaining 120 were used for model testing.

#### 4.1.2. UFBA-UESC Dental Dataset

The diagnostic imaging center of the Southwest State University of Bahia (UFBA-UESC) dental dataset [39] contained 1500 images for the classification and segmentation of teeth in panoramic X-ray images. After data augmentation, there were 5400 images; of these, 4320 were used for model training, 540 were used for validation and the remaining 540 were used for model testing.

### 4.2. Experimental Settings

To ensure a fair comparison, all the experiments were performed under identical conditions. The Keras framework and an NVIDIA GeForce RTX 3090 graphics processing unit (GPU) were used for model training. GPU implementation was performed using the NVIDIA CUDA deep neural network library (cuDNN). For comparison, we obtained the codes of existing models and altered the parameters such as network layers to maintain the same experimental environment across all methods. All existing and proposed models were trained using the input dimensions of 256 × 256 × 3 and produce output dimensions of 256 × 256 × 1 with the following hyperparameters; we selected Adam as an optimizer for stochastic gradient descent, which optimizes the objective information of the model. The batch size was set as 4, the learning rate was 0.001, the validation split was 0.2, the maximum number of iterations was 100, and the total number of network layers was set to 3. Encoder and decoder blocks of the conventional methods used *k* × *k* sized convolution kernels with *k* = 1 and 3, while the proposed method used a spatial group convolution with *k* × 1 and 1 × *k* kernels where *k* = 1, 3, 5, and 7. In addition, all the comparison models used a max-pooling operation 2 × 2 with a stride of 2. However, the proposed method used max-pooling operations 2 × 2, 4 × 4, and 8 × 8 with a stride of 2. The Sigmoid function was used as an output classifier for all models. The segmentation performance of the proposed method was evaluated using accuracy (*ACC*). The accuracy was the percentage of pixels correctly categorized in the input image and was defined as in (5). However, a higher accuracy value does not always imply a superior ability for image segmentation. To better correlate the performance of the obtained metric value against the obtained segmentation outputs, we also evaluated the proposed method using two alternative metrics, such as dice similarity coefficient (*DSC*) and Jaccard index (*JI*), which were defined as (6) and (7).

(5) $$ACC=\frac{TP+TN}{TP+TN+FP+FN}$$

(6) $$DSC=\frac{2.TP}{2.TP+FP+FN}$$

(7) $$JI=\frac{TP}{TP+FP+FN}$$

where *TP* (*TN*) represents the number of pixels correctly classified as positive (negative), and *FP* (*FN*) represents the number of pixels incorrectly classified as positive (negative). The *DSC*, *JI*, and *ACC* metrics were used to assess the segmentation accuracy by determining whether the pixels were correctly classified as positive or negative. The evaluation metrics Dice and JI are highly comparable. Both *DSC* and *JI* range from 0 to 1, with 1 denoting the most similarity between predicted and true values. We also measured the processing times of existing and the proposed methods by summing total training time and average test time. Total training time is the total required time for a model to train and average test time indicates the average times required to produce segmentation maps on the test images by the given trained model.

### 4.3. Results and Discussions

The proposed method was compared with U-Net [10], M-Net [11], CE-Net [13], M-SegNet [14], RA-UNet [15], nnU-Net [17], and CMM-Net [16]. Table 1 presents the experimental results for the proposed method and conventional methods with regard to the segmentation accuracy for skin and tooth segmentation tasks. The proposed method achieved a significantly higher segmentation accuracy than the other methods according to the *DSC*, *JI*, and *ACC* metrics. As shown in Table 1, U-Net achieved a mean segmentation accuracy of 0.92 for skin lesions and 0.93 for tooth segmentation with 1.9 M parameters. The performance is attributed to the skip connections, which made the semantic level of the encoder feature maps closer to that of the feature maps waiting in the decoder. M-Net and CE-Net consistently outperformed U-Net, with CE-Net generating a large number of model parameters (approximately 7 M). M-SegNet with deep supervision (5 M parameters) outperformed both M-Net and CE-Net regarding the *DSC*, with average improvements of 3% and 2%, respectively. Although U-Net and M-Net exhibited lower computational complexity, they achieved lower *DSC* values for both tooth and lesion segmentation. CE-Net and M-SegNet exhibited comparable similarity scores to the proposed method but had higher computational complexities.

**Figure 4:**
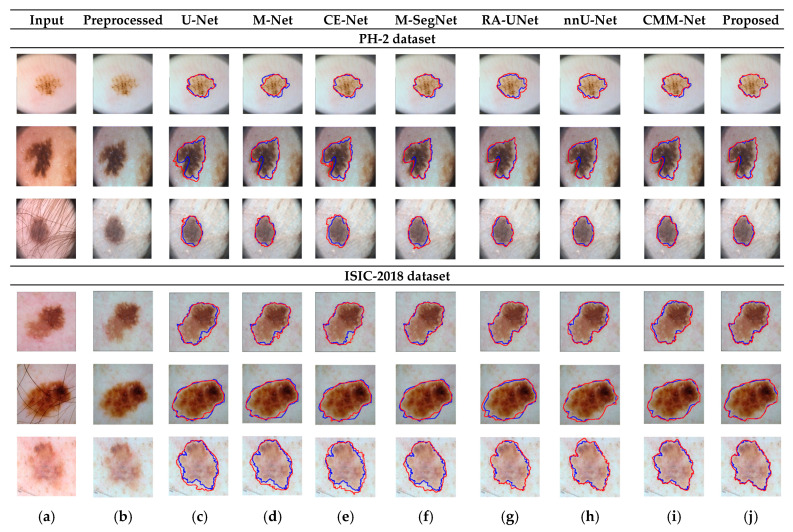
Qualitative comparison of the proposed method and conventional methods for a skin-lesion dataset. From left to right: (**a**) original input images; (**b**) preprocessed images; (**c**–**j**) input images with the overlay of ground truth (blue contour) and predicted outputs (red contour) indicating the segmentation results obtained by U-Net, M-Net, CE-Net, M-SegNet, RA-UNet, nnU-Net, CMM-Net, and the proposed method, respectively.

**Figure 5:**
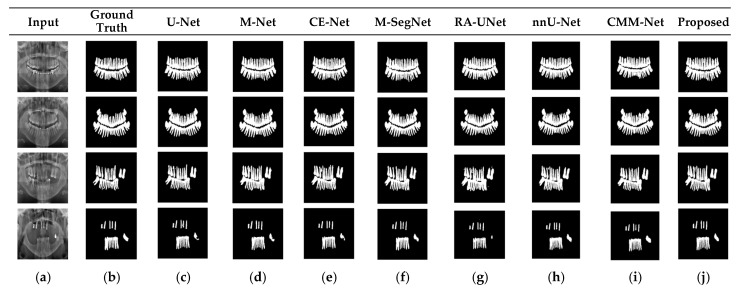
Qualitative comparison of the proposed method and existing methods for the UFBA-UESC dental dataset. From left to right: (**a**) original input image; (**b**) ground-truth segmentation map; (**c**–**j**) segmentation results obtained using U-Net, M-Net, CE-Net, M-SegNet, RA-UNet, nnU-Net, CMM-Net, and the proposed method, respectively.

RA-UNet trains a 2D network, followed by a 3D network, resulting in a computationally expensive network with poorly segmented results. Similarly, the nnU-Net and CMM-Net architectures focus on effective model training and ignore architectural changes that are crucial for accuracy improvements. Compared with the conventional methods, the proposed method achieved higher *ACC* and *DSC* values with a moderate number of model parameters, exhibiting the best performance. The improved performance of the proposed method was due to the combination of the guided block and feature-aggregation modules. The *HFA* in the proposed method acts as the backbone for discriminative feature extraction with *MFA*-based long residuals, aggregates the network layers, and allows for gradients to flow through the network. The *MFA*-based modules such as *CEM*, *IM*, and *LEM* use different kernel sizes *k* = 1, 3, 5, and 7 and extract the features information at multiscale levels. Aggregating this multiscale information helps to maintain the output feature resolutions. It is different from conventional U-net, M-net, and CE-net methods, which use the low-resolution feature information obtained from fixed-size kernel operations. In addition, although the proposed method uses larger kernel sizes *k* = 5 and 7, it can be computed with smaller spatial kernel operations (1 × *k* and *k* × 1) with the same kernel effect at the cost of significantly reduced model parameters. The *MFA* block captures local feature information, whereas *MFA*-based long residuals help the model learn latent features with reference to the input layer, and thus prevent the information loss which usually occurs in the non-residual-based deep architectures such as nnU- Net and M-SegNet. The proposed method employing the *HFA* and *MFA* obtained a hierarchically structured, multiscale representation and achieved a higher segmentation accuracy than the other methods with fewer parameters. Furthermore, the feature maps of the encoder are directly received by the decoder in conventional methods, while the proposed method performs multilevel convolution through the guided block, which can improve the boundary representation of the skin lesions and teeth, and refines the misaligned boundaries with a multilevel spatial kernels. Figure 4 shows the segmentation results for a skin lesion obtained using the proposed and conventional methods with the PH2 and ISIC-18 datasets. Figure 4a,b show the original input and preprocessed skin-lesion images, respectively. The input images with an overlay of ground truth (red outline) and predicted lesion segmentation output (blue outline) for U-Net, M-Net, CE-Net, M-SegNet, RA-UNet, nnU-Net, CMM-Net, and the proposed methods are shown in Figure 4c–j. Similarly, Figure 5 shows the segmentation results for a tooth from the UFBA-UESC dental dataset obtained using the proposed method and conventional methods. As shown in Figure 4 and Figure 5, U-Net, M-Net, and CE-Net misclassified some parts of the background skin as lesions and failed to accurately distinguish between normal and lesion pixels. The M-SegNet and RA-UNet models produced segmentation results with more noise in the dermoscopic images. Compared with the proposed method, the outputs of both nnU-Net and CMM-Net exhibited a significant variance from the ground-truth segmentation map. Furthermore, the proposed method produced subjectively well-segmented results by accurately capturing lesion contours and sharp tooth boundary transitions.

### 4.4. Ablation Study

The proposed framework is composed of three main components: an *MFA*, an *HFA*, and a guided block for the segmentation of medical images. To verify the contributions of each component, we performed ablation studies with skin-lesion datasets using four variants of the proposed model. The details of the proposed architecture variants are presented in Table 2. Variation 1 was the basic encoder–decoder network with a 3 × 3 convolution kernel for feature extraction, followed by 2 × 2 max pooling and 2 × 2 upsampling layers at the encoder and decoder, respectively. Feature maps were copied from the encoder to the decoder via skip connections. Variation 2 comprised the proposed *HFA* module, which replaced the typical 3 × 3 convolution in variation 1. Similarly, for both the encoder and decoder stages, Variation 3 included a normal 3 × 3 convolution combined with *MFA*-based long residual connections. In addition, the proposed guided block was incorporated into Variation 1 to obtain attention-based skip connections, which resulted in Variation 4. Finally, the proposed method (with all the components) was tested for comparison. According to the experimental results, the Variation 1 model with the basic encoder–decoder architecture exhibited the fewest model parameters and low *DSC* and *ACC* values. For Variation 3 with multiscale-based long residuals, the *ACC* and *DSC* were improved by 0.5% and 1%, respectively, and the number of parameters was slightly reduced (approximately 1.2 M) compared with Variation 1. Variation 2, in which the standard 3 × 3 convolutions were replaced with the *HFA*, exhibited *ACC* and *DSC* improvements of 1.5% and 2%, respectively, compared with Variation 3. Hence, the *HFA* was advantageous compared to the *MFA*, achieving higher accuracy and similarity scores. Furthermore, the guided block, which produced guided skip connections, contributed to the maximum results compared with the individual *HFA*- and *MFA*-based Variations 2 and 3.

However, the segmentation outcomes obtained by the individual components were inferior to those obtained by the proposed method, which integrated all the components. Furthermore, the combination of *HFA*, *MFA* and guided attention led to the optimal performance: 97% *ACC*, 95% *DSC*, 90% JI, and 3 M parameters. These results represent an improvement of 6% in the *DSC* compared with the baseline encoder–decoder architecture, demonstrating the superiority of the proposed combination of aggregation modules and guided blocks to the individual components.

We performed a Wilcoxon rank-sum test to compare the proposed method with existing methods to verify a statistically significant difference in the *DSC* scores. Table 3 presents the *p*-values from the Wilcoxon rank-sum tests for the *DSC* metric obtained using the PH2 dataset. As shown in Table 3, the proposed method with higher *DSC* scores for most of the skin image samples attains a *p*-value of less than 0.05 (5%), which indicates that the proposed method outperforms the other conventional methods.

**Figure 6:**
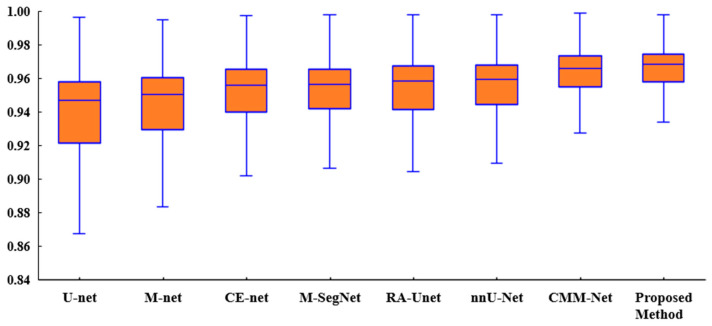
Box plot of the accuracies of the proposed method and existing methods for the UFBA-UESC dental dataset.

Figure 6 presents a box plot of the *ACC* metric for the proposed method and existing methods for the tooth segmentation task. As shown, the proposed method achieved better results than the state-of-the-art methods with regard to the statistical dispersion of the samples in the dental dataset. This indicates that the proposed method can achieve more consistent results regarding segmentation accuracy.

## 5. Conclusions

We propose a novel and effective multiscale and hierarchical feature-aggregation network for the segmentation of medical images. It contains two feature-aggregation modules—*MFA* and *HFA*—to better fuse information across the network layers. In addition, guided skip connections are used to transfer features from the encoder to the decoder for recovery of the spatial information lost during downsampling. Experimental results obtained using publicly available datasets indicated that the proposed model has a good generalization ability and can, therefore, be applied to various medical image segmentation tasks. For further performance improvements, we will consider transfer learning and cascading structures for medical-image segmentation. In the future, it would be important to modify the structures of the current convolutional neural network models to construct more effective systems. Furthermore, well-defined architectures that can work with various types of health data are required to solve complicated challenges in the area of healthcare. In terms of explainable artificial intelligence, it is also critical to integrate deep learning models in distributed systems, which can drastically reduce processing times.

## Figures and Tables

**Table 1:** Comparison of the segmentation accuracy among the proposed method and conventional ones.

Models	PH2 [38]			Processing Time	ISIC-2018 [37]			Processing Time	UFBA-UESC [39]			Processing Time	# of Parameters
	ACC	DSC	JI		ACC	DSC	JI		ACC	DSC	JI		
**U-Net** [10]	0.92	0.89	0.80	9.1 min	0.90	0.84	0.72	109.2 min	0.93	0.91	0.83	64.2 min	1,946,881
**M-Net** [11]	0.93	0.90	0.82	10.4 min	0.91	0.85	0.74	128.5 min	0.94	0.92	0.85	79.3 min	2,337,505
**CE-Net** [13]	0.94	0.91	0.84	36.2 min	0.93	0.86	0.75	188.9 min	0.95	0.92	0.85	168.9 min	7,356,929
**M-SegNet** [14]	0.96	0.93	0.87	19.2 min	0.94	0.86	0.75	159.5 min	0.96	0.93	0.87	123.6 min	5,468,932
**RA-UNet** [15]	0.94	0.90	0.82	12.6 min	0.93	0.86	0.75	137.8 min	0.95	0.90	0.83	84.2 min	2,935,505
**nnU-Net** [17]	0.95	0.91	0.84	91.9 min	0.94	0.87	0.77	767.5 min	0.95	0.91	0.84	591.3 min	28,285,984
**CMM-Net** [16]	0.96	0.94	0.87	45.3 min	0.95	0.88	0.79	309.6 min	0.96	0.92	0.85	268.4 min	10,252,673
**Proposed**	**0.97**	**0.95**	**0.90**	17.8 min	**0.95**	**0.89**	**0.80**	127.1 min	**0.97**	**0.94**	**0.89**	108.5 min	**3,165,825**

**Table 2:** Comparison of the proposed method and its variations for skin-lesion test images.

Model	ACC	DSC	JI	Basic	HFA	MFA with Residual	Guided Block	# of Parameters
Variation 1	0.926	0.893	0.809	✓	×	×	×	1,946,881
Variation 2	0.947	0.923	0.865	×	✓	×	×	2,045,089
Variation 3	0.932	0.903	0.828	✓	×	✓	×	1,209,345
Variation 4	0.956	0.935	0.885	✓	×	×	✓	2,119,809
Proposed	**0.972**	**0.951**	**0.903**	✓	✓	✓	✓	**3,165,825**

**Table 3:** *p*-values from Wilcoxon rank-sum tests for the *DSC* metric using the PH2 dataset.

**Metric**	**U-Net** **vs.** **Proposed**	**M-Net** **vs.** **Proposed**	**CE-Net** **vs.** **Proposed**	**M-SegNet** **vs.** **Proposed**	**RA-UNet** **vs.** **Proposed**	**nnU-Net** **vs.** **Proposed**	**CMM-Net** **vs.** **Proposed**
*DSC*	0.010	0.015	0.024	0.031	0.033	0.035	0.038

## Data Availability

Not applicable.
